# Time to ART Initiation among Patients Treated for Rifampicin-Resistant Tuberculosis in Khayelitsha, South Africa: Impact on Mortality and Treatment Success

**DOI:** 10.1371/journal.pone.0142873

**Published:** 2015-11-10

**Authors:** Johnny Flippie Daniels, Mohammed Khogali, Erika Mohr, Vivian Cox, Sizulu Moyo, Mary Edginton, Sven Gudmund Hinderaker, Graeme Meintjes, Jennifer Hughes, Virginia De Azevedo, Gilles van Cutsem, Helen Suzanne Cox

**Affiliations:** 1 Médecins sans Frontières, Khayelitsha, Cape Town, South Africa; 2 Médecins sans Frontières, Luxembourg, Luxembourg; 3 Human Sciences Research Council, HIV/AIDS, STIs and TB programme, Cape Town, South Africa; 4 International Union against TB and Lung Disease, Paris, France; 5 School of Public Health, Faculty of Health Sciences, University of the Witwatersrand, Johannesburg, South Africa; 6 University of Bergen, Bergen, Norway; 7 Institute of Infectious Disease and Molecular Medicine and Department of Medicine, University of Cape Town, Cape Town, South Africa; 8 City of Cape Town Department of Health, Cape Town, South Africa; 9 Centre for Infectious Disease Epidemiology and Research, University of Cape Town, Cape Town, South Africa; 10 Division of Medical Microbiology and Institute of Infectious Disease and Molecular Medicine, University of Cape Town, Cape Town, South Africa; CEA, FRANCE

## Abstract

**Setting:**

Khayelitsha, South Africa, with high burdens of rifampicin-resistant tuberculosis (RR-TB) and HIV co-infection.

**Objective:**

To describe time to antiretroviral treatment (ART) initiation among HIV-infected RR-TB patients initiating RR-TB treatment and to assess the association between time to ART initiation and treatment outcomes.

**Design:**

A retrospective cohort study of patients with RR-TB and HIV co-infection not on ART at RR-TB treatment initiation.

**Results:**

Of the 696 RR-TB and HIV-infected patients initiated on RR-TB treatment between 2009 and 2013, 303 (44%) were not on ART when RR-TB treatment was initiated. The median CD4 cell count was 126 cells/mm^3^. Overall 257 (85%) patients started ART during RR-TB treatment, 33 (11%) within 2 weeks, 152 (50%) between 2–8 weeks and 72 (24%) after 8 weeks. Of the 46 (15%) who never started ART, 10 (21%) died or stopped RR-TB treatment within 4 weeks and 16 (37%) had at least 4 months of RR-TB treatment. Treatment success and mortality during treatment did not vary by time to ART initiation: treatment success was 41%, 43%, and 50% among patients who started ART within 2 weeks, between 2–8 weeks, and after 8 weeks (p = 0.62), while mortality was 21%, 13% and 15% respectively (p = 0.57). Mortality was associated with never receiving ART (adjusted hazard ratio (aHR) 6.0, CI 2.1–18.1), CD4 count ≤100 (aHR 2.1, CI 1.0–4.5), and multidrug-resistant tuberculosis (MDR-TB) with second-line resistance (aHR 2.5, CI 1.1–5.4).

**Conclusions:**

Despite wide variation in time to ART initiation among RR-TB patients, no differences in mortality or treatment success were observed. However, a significant proportion of patients did not initiate ART despite receiving >4 months of RR-TB treatment. Programmatic priorities should focus on ensuring all patients with RR-TB/HIV co-infection initiate ART regardless of CD4 count, with special attention for patients with CD4 counts ≤ 100 to initiate ART as soon as possible after RR-TB treatment initiation.

## Background

The emergence of rifampicin-resistant tuberculosis (RR-TB) challenges the success of national TB control programs and the survival of patients with the disease [1]. RR-TB, or TB with any resistance to rifampicin, includes rifampicin mono-resistant tuberculosis, multidrug-resistant tuberculosis (MDR-TB), and extensively resistant tuberculosis (XDR-TB) [1]. South Africa has high dual burdens of both RR-TB and human immunodeficiency virus (HIV). In 2013, 26,023 laboratory-confirmed RR-TB cases were reported in South Africa with only 10,663 (41%) reported to have been initiated on second-line anti-tuberculosis treatment [1]. In 2012 it was estimated that 6.4 million people (12%) of the total South African population were living with HIV [2]. According to the 2014 World Health Organization Global TB Report, 62% of all TB cases in South Africa are co-infected with HIV and only 66% of these received antiretroviral therapy (ART) during TB treatment [1].

Patients with MDR-TB who are HIV-infected have been reported to have high early mortality [3,4]. In KwaZulu Natal, South Africa, one year mortality was more than 70%, with 30% of deaths occurring within 30 days of MDR-TB diagnosis [5]. Prior to widespread ART provision in South Africa, HIV-infected MDR-TB patients had a two-fold mortality when compared to HIV uninfected patients [4]. Improved survival was reported more recently from our setting in Khayelitsha, South Africa, although early mortality remains substantial, particularly among HIV-infected patients [6]. More rapid provision of ART might be expected to reduce this excess mortality [7,8].

In KwaZulu Natal, among HIV co-infected XDR-TB patients, provision of ART at any point during second-line treatment was associated with improved survival, particularly for patients with CD4 counts ≤ 200 cells/mm^3^ [3]. Similarly, ART provision during treatment was associated with improved 12-month survival among XDR-TB patients in four provinces across South Africa [9].

While it is evident that the initiation of ART at some point during RR-TB treatment is beneficial, evidence supporting the optimal time to initiate ART is limited. Second-line treatment for RR-TB is less well tolerated than first-line TB treatment with higher rates of adverse events [10,11]. Given the potential for additive toxicity with ART [12], it may be beneficial to delay ART initiation until second-line TB treatment is tolerated.

Current clinical guidelines in South Africa regarding the appropriate time to initiate ART are primarily based on data from patients with drug-susceptible TB [13–15]. In these randomized trials, ART initiation within 2–4 weeks was associated with improved survival in those patients with low CD4 counts (<50 cells/mm3). Since 2008, most TB and HIV guidelines have recommended ART for all RR-TB patients within South Africa. There is, however, a lack of consensus with respect to timing of ART initiation for RR-TB patients (Table 1).

**Table 1:** South African guidelines and recommendations for antiretroviral treatment (ART) initiation for drug sensitive- and rifampicin-resistant tuberculosis patients 2008–2014.

Year	Source	Recommendation
2008	Guidelines–Antiretroviral therapy in adults: Southern African HIV Clinicians Society. [21]	**TB diagnosed before starting ART**
		CD4 count <200: commence ART after it is clear that the patient’s TB symptoms are improving and that TB therapy is tolerated (between 2 and 8 weeks).
		CD4 count 200–350: delay ART until after the intensive phase of TB therapy (2 months) unless the patient has other serious HIV-related illness.
		CD4 count >350: defer ART.
		**No specific recommendation for RR-TB**
2009	Draft: Management of drug-resistant tuberculosis: policy guidelines. SANDOH. [22]	**Initiation of ART at the earliest opportunity is encouraged**
		If CD4 count 350 and no other HIV-related symptoms: Start DRTB treatment. Assess the need for ART after completing treatment, using CD4 and clinical criteria.
		If CD4 count <350: Delay ART until after 1 month of DR-TB treatment.
		If CD4 count of <50/ mm^3^ or presence of other serious HIV illness: Introduce ART as soon as the patient is stabilized on DR-TB treatment, preferably within the first month.
2009	SA National Tuberculosis Management Guidelines. SANDOH. [23]	**TB diagnosed before starting ART**
		CD4 count 50–200: Delay ART for two months (until intensive phase of TB therapy complete).
		CD4 count of <50 or other serious HIV illness: Introduce ART regimen above as soon as the client is stabilized on TB therapy (at least 2 weeks between starting TB therapy and starting ART).
		**No specific recommendation for RR-TB**
2010	The South African antiretroviral treatment guidelines. SANDOH. [24]	Require fast track (i.e. ART initiation within 2 weeks of being eligible–MDR-/XDR-TB irrespective of CD4 count.
2011	Management of Drug-resistant tuberculosis: policy guidelines. SANDOH. [28]	**The National guidelines on the use of antiretroviral therapy need to be considered in conjunction with the content of this chapter**
		All HIV positive, MDR- and XDR-TB patients are eligible to start ART irrespective of CD4 cell count. Moreover, these patients must be fast-tracked **(ART initiation within 2 weeks of being eligible) for the initiation of ART**.
		All patients must be started on ART irrespective of CD4 cell count. Moreover the initiation of ART must be fast tracked as soon the DR-TB treatment is tolerated preferably within first month of treatment
2012	Guidelines–Antiretroviral therapy in adults: Southern African HIV Clinicians Society. [38]	**Starting ART in patients with TB:** Decisions regarding the timing of ART in patients with TB should be made on the basis of the CD4 count:
		CD4 count ≤50: ART should be regarded as urgent, and the aim should be to start therapy after 2 weeks of TB treatment.
		CD4 count >50: ART should be delayed until after the intensive phase of TB treatment (2 months) unless the patient has other serious HIV-related conditions (e.g. Kaposi’s sarcoma or HIV encephalopathy.
		**No specific recommendation for RR-TB**
2013	The South African antiretroviral treatment guidelines, SANDOH. [17]	**Starting ART in patients with TB**
		ART indicated irrespective of CD4 count: All types of TB (In patients with TB/HIV drug resistant or sensitive TB, including extra pulmonary TB).
		Patients with TB/HIV co morbidity with CD4 count <50 require fast track (i.e. ART initiation within 7 days of being eligible).
		In general, ART should be initiated as soon as the patient is tolerating their TB therapy.
January 2013	Management of Drug-resistant tuberculosis: policy guidelines, updated. SANDOH. [16]	All HIV-positive TB, MDR- and XDR-TB patients are eligible to start ART irrespective of CD4 cell count. Furthermore, these patients must be fast-tracked (ART initiation within 2 weeks of being eligible) for the initiation of ART.
2014	Guidelines–Antiretroviral therapy in adults: Southern African HIV Clinicians Society. [39]	**Starting ART in patients with TB**: Decisions regarding the timing of ART in patients with TB should be made on the basis of the CD4 count:
		CD4 count ≤50: ART should be regarded as urgent, with the aim to start therapy 2 weeks following the commencement of TB treatment.
		CD4 count >50: ART can be delayed until 8 weeks after starting TB treatment, but no later. However, if the patient has other WHO stage 4 conditions, ART should also be initiated 2 weeks after TB treatment is started.
		**No specific recommendation for RR-TB**
2014	National Tuberculosis Management Guidelines, SANDOH. [40]	**TB diagnosed before ART**
		Patients with CD4 count <50: Fast track- start ART within 2 weeks after starting TB treatment.
		Patients with CD4 count >50: Start ART 2–8 weeks after starting TB treatment.
		Patients with TB meningitis (irrespective of CD4 count: Defer ART until 8 weeks after starting TB treatment
		**No specific recommendation for RR-TB**
SANDOH: South African National Department of Health		

Guidance for ART initiation is available in the 2013 South African National Drug-Resistant TB guidelines and the 2013 South African ART guidelines [16,17]. The National Drug-Resistant TB guidelines provide conflicting information; firstly they state that the optimal time to ART is not known and that ART initiation depends on a careful calculation of risk and benefits for each patient [16]. However, the guidelines advise that all HIV-infected TB patients, including RR-TB patients, are eligible for ART initiation irrespective of CD4 cell count, and should be “fast-tracked” for ART initiation within 2 weeks of being eligible; eligibility criteria are not explicitly outlined. Thereafter, they suggest that ART be started within the first month of RR-TB treatment or when RR-TB treatment is tolerated [16]. In contrast the 2013 South African ART guidelines suggest ART “fast-tracking” (within 7 days) for all TB patients with CD4 < 50 cells/mm^3^ [17]; for the remainder, these guidelines suggest 2–8 weeks of TB therapy before commencing ART [17]. The inconsistencies across national guidelines are likely to lead to uncertainty amongst clinicians regarding appropriate time for ART initiation among RR-TB patients, along with the potential for significant delays and missed opportunities for ART initiation in co-infected patients.

Due to the lack of direct evidence regarding the timing of ART initiation for RR-TB patients, we aimed to describe time to ART initiation among RR-TB patients in a community-based RR-TB programme in Khayelitsha, Cape Town. Additionally, we aimed to assess associations between time to ART initiation and the patient outcomes of treatment success and mortality.

## Methods

### Ethical approval

Ethical approval was received from the University of Cape Town Human Research Ethics Committee (540/2010) for the evaluation of the decentralized programme. Additional ethical approval specific to this study was received from the Ethics Advisory Group of the International Union against Tuberculosis and Lung Disease, Paris, France and the MSF Ethics Review Board, Geneva, Switzerland. All routine programme data used for analysis was anonymized and de-identified as informed consent was not required for this retrospective cohort study.

### Design

We conducted a retrospective cohort study of HIV-infected RR-TB patients not on ART at initiation of second-line anti-tuberculosis treatment. Our outcome measurements were time to ART initiation and RR-TB treatment outcomes.

### Setting and population

The study was conducted in Khayelitsha, the largest township in South Africa’s Western Cape Province, where government health services in collaboration with Médecins Sans Frontières developed a decentralized model of RR-TB care. This decentralized model was integrated into the existing national TB program in 2008 and handed over to government for management in 2011 [6,18]. Khayelitsha has among the highest burdens of TB, including RR-TB, and HIV infection in the country and globally. In 2011, HIV antenatal prevalence was estimated to be 37% and the TB case notification rate was at least 1,500 per 100,000 people a year. More than 200 RR-TB patients are diagnosed every year, of which 70% are HIV co-infected [6,18,19]. RR-TB diagnosis and treatment services are provided through 10 primary health care clinics and one secondary level hospital; integrated TB and HIV services are offered to a target population of an estimated 400,000 people, half of whom live in informal settlements [20].

Diagnosis, registration, and treatment initiation for RR-TB was conducted according to the National Drug-Resistant TB guidelines at the time of the study. All patients diagnosed with RR-TB started on a standardized second- line TB treatment regimen, with subsequent adjustment of treatment if any second-line drug resistance was detected. At the start of the study period in 2009, the treatment regimen in Khayelitsha included ofloxacin, kanamycin, ethambutol, ethionamide, and pyrazinamide. Cycloserine was added from September 2009, and was replaced by terizidone in April 2010; ofloxacin was also replaced by moxifloxacin in September 2009. Since 2008, all HIV-infected RR-TB patients have been eligible for ART, regardless of CD4 count; the aim has been to initiate ART as soon as possible, but no specific guidance was available on the timing of ART. First-line ART included zidovudine (or stavudine), lamivudine, and efavirenz (or nevirapine) [16,17,21–25]. More recently, tenofovir became one of the preferred non-nucleoside reverse transcriptase inhibitor options, but was mostly avoided during aminoglycoside treatment [16,17]. Patients received individual structured RR-TB counselling as well as HIV counselling and testing at diagnosis and start of RR-TB treatment. The study included all RR-TB and HIV co-infected patients starting RR-TB treatment in Khayelitsha between January 2009 and December 2013 who were not on ART at the time of RR-TB treatment initiation.

### Data variables, sources, and statistical analysis

Data was sourced from the Khayelitsha RR-TB database and cross checked with medical records for confirmation of data specific to this study. The Khayelitsha RR-TB database was established in 2008 for evaluation of the decentralized model of care and all data was collected prospectively from 2008 [6]. The STROBE guidelines on the reporting for observational studies were followed [26]. Data meeting the inclusion criteria were extracted into an Excel database (Microsoft, Redmond, WA, USA). CD4 count was defined as CD4 result available at time at RR-TB diagnosis or within one month of RR-TB treatment start. Final RR-TB treatment outcomes were defined according to the South African National Guidelines, which are in line with the World Health Organization outcome definitions, with the exception of treatment failure, where failure to convert positive cultures to negative within 6–8 months constitutes failure of treatment [22,27,28]. The time from RR-TB treatment start to start of ART was calculated in days and then categorised as ≤ 2 weeks, 2–8 weeks and > 8 weeks; time windows were derived from the guidelines described in Table 1.

Data was analysed using STATA/IC 12 software (Stata Corp, College Station, TX, USA) and descriptive statistics were used to report demographic, clinical, and immunological characteristics of patients. Final treatment outcome proportions were compared by chi-square tests; a p–value < 0.05 was considered significant. Time to ART initiation was shown using a Kaplan Meier curve. To assess the impact of time to ART initiation on mortality, a multivariate Cox regression analysis was performed. Censoring for both analyses was done at loss from RR-TB treatment (previously defined as lost to follow-up) and transfer out. Data was administratively censored as of 30 June 2014, when the analysis was conducted. Factors significant (p<0.05) from univariate analysis and those previously determined to be associated with mortality among RR-TB patients were included in the model.

## Results

During the study period between January 2009 to December 2013, 982 laboratory-confirmed RR-TB patients from Khayelitsha initiated second-line RR-TB treatment. Among these, 71% (696/982) were HIV positive and 56% (393/696) of these were on ART at start of RR-TB treatment, leaving 303 RR-TB and HIV positive patients not on ART at the time of starting second-line TB treatment for inclusion in the analysis. Demographic, clinical, and immunological characteristics are shown in Table 2. The median CD4 count was 126 cells/mm^3^ and 152 (41%) had a CD4 count ≤100 cells/mm^3^.

**Table 2:** Demographic, clinical and immunological characteristics of patients starting treatment for rifampicin-resistant tuberculosis in patients co-infected with HIV not receiving antiretroviral treatment, in Khayelitsha, South Africa 2009–2013.

Variable	ART Started^a^				
	≤2 weeks	2–8 weeks	>8 weeks	Never	Total
	n (%)	n (%)	n (%)	n (%)	
**Total**	33 (11)	152 (50)	72 (24%)	46 (15)	303 (100)
Median time(weeks) (IQR)	1.5 (1–2)	4 (3–6)	15 (10–20)		
**Gender**					
Male	12 (36)	70 (46)	30 (42)	26 (57)	138 (46)
Female	21 (64)	82 (54)	42 (58)	20 (43)	165 (54)
**Age group (years)**					
0–25	5 (15)	26 (17)	16 (22)	7 (15)	54 (18)
26–30	8 (24)	39 (26)	10 (14)	7 (15)	64 (21)
31–35	4 (12)	32 (21)	18 (25)	11 (24)	65 (21)
36–40	7 (21)	31 (20)	13 (18)	13 (28)	64 (21)
41+	9 (28)	24 (16)	15 (21)	8 (18)	56 (19)
Median age (IQR)	33 (27–41)	32 (28–37)	33 (26–39)	24 (29–40)	33 (18–55)
**TB treatment status**					
New	11 (33)	62 (41)	26 (36)	15 (33)	114 (37)
Retreatment	22 (67)	90 (59)	46 (67	30 (65)	188 (62)
Unknown	0 (0)	0 (0)	0 (0)	1 (2)	1 (0)
**CD4 count (cells/mm3)**					
0–100	16 (49)	69 (45)	18 (25)	22 (48)	125 (41)
100–200	9 (27)	38 (25)	11 (15)	9 (19)	67 (22)
200–300	3 (9)	21 (14)	11 (15)	7 (15)	42 (14)
>300	5 (15)	24 (16)	32 (45)	8 (18)	69 (23)
Median cd4 (IQR)	101 (38–153)	111 (48–217)	274 (106–460)	112 (52–229)	126 (54–280)
**RR-TB** ^**b**^ **profile**					
R-mono^c^	7 (21)	22 (14)	20 (28)	11 (24)	60 (20)
GXP RR^d^ only	1 (3)	6 (4)	2 (3)	1 (2)	10 (3)
MDR^e^	21 (64)	105 (69)	48 (66)	28 (61)	202 (67)
MDR+SLDres^f^	4 (12)	19 (13)	2 (3)	6 (13)	31 (10)
**Type of TB disease**					
Smear positive PTB^g^	13 (39)	65 (43)	36 (50)	19 (41)	133 (44)
Smear negative PTB^g^	17 (52)	73 (48)	33 (47)	21 (46)	144 (48)
EPTB^h^	2 (6)	6 (4)	1 (1)	2 (4)	11 (4)
Both PTB^g^ & EPTB^f^	1 (3)	4 (3)	1 (1)	0 (0)	6 (2)
Unknown	0 (0)	4 (3)	1 (1)	4 (9)	9 (3)
**Site of treatment initiation**					
Primary care^k^	23 (70)	124 (81)	63 (88)	31 (67)	241 (80
Khayelitsha district hospital	0 (0)	7 (5)	3 (4)	3 (7)	13 (4)
Tertiary hospital outside Khayelitsha	10 (10)	21 (14)	6 (8)	6 (26)	49 (16)
**Year started treatment**					
2009	8 (9)	23 (26)	28 (31)	14 (34)	73
2010	7 (12)	26 (46)	15 (26)	9 (16)	57
2011	6 (10)	33 (55)	13 (22)	8 (13)	60
2012	8 (23)	36 (60)	7 (12)	9 (25)	60
2013	4 (8)	34 (64)	9 (17)	6 (11)	53

Percent (column)

^a^ART: Antiretroviral treatment

^b^RR-TB: Rifampicin-resistant tuberculosis

^c^R-mono: Rifampicin mono resistance

^d^GXP: Gene-Xpert MTB/RIF rifampicin resistance only

^e^MDR: Multi-drug resistant tuberculosis with no confirmed second-line resistance

^f^SLDres: second-line drug resistance

^g^PTB: Pulmonary TB

^h^EPTB: Extra-pulmonary TB

^k^Primary care: treatment initiation at clinic and local sub-acute care facility

Percent (Row)

Year RR-TB^b^ treatment started

**Fig 1:**
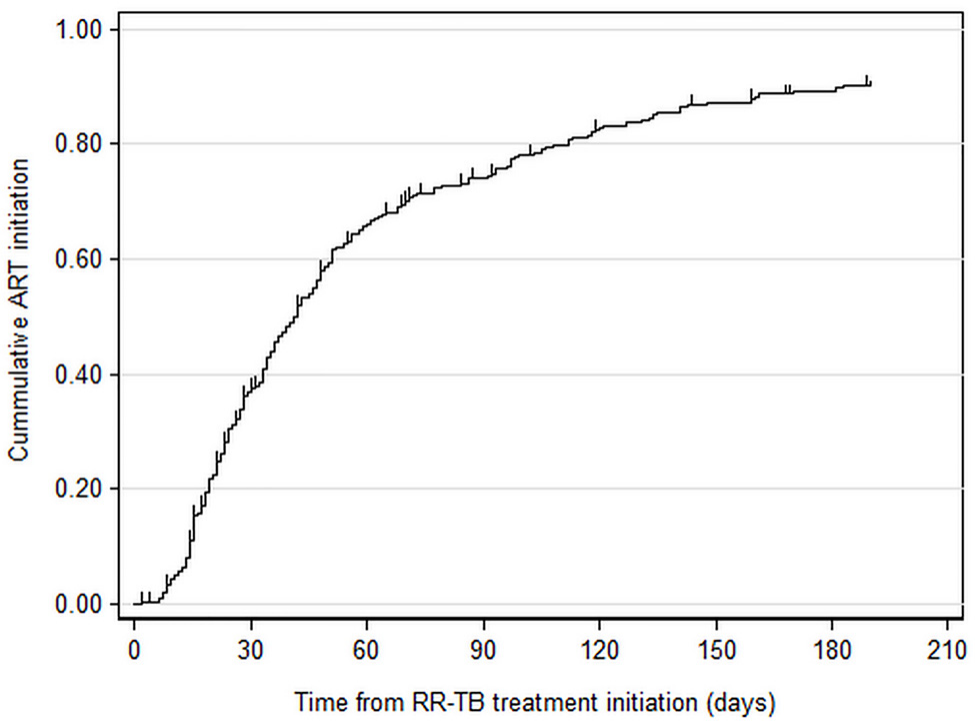
Kaplan-Meier plot of time to antiretroviral treatment initiation for HIV infected rifampicin resistant tuberculosis patients.

Overall 257 (85%) of patients were recorded as initiating ART; 33 (11%) started ART within 2 weeks, 152 (50%) within 2–8 weeks, and 72 (24%) started after 8 weeks (Table 2). Time to ART initiation is shown in a Kaplan-Meier graph (Fig 1). Of all those patients who initiated ART, respectively, 35%, 65%, and 75% of patients had been initiated on ART at 30, 60, and 90 days.

Among the 46 patients who never initiated ART, nine (20%) died, one experienced loss from treatment, and three were transferred out within 4 weeks of starting second-line treatment. Among the nine patients with early mortality and no ART, four (44%) had CD4 counts less than 100 cells/mm^3^. At 8 weeks post RR-TB treatment initiation, 28 patients known to be in clinical care and eligible for ART (median CD4 count 116 cells/mm^3^) were not yet initiated; by 16 weeks, 16 patients remaining in care and eligible (median CD4 count 132 cells/mm^3^) were still not initiated on ART. At 6 months after RR-TB treatment initiation, 66% of the 12 patients remaining in care but not initiated on ART had already converted their positive sputum cultures to negative, indicating good retention in care and representing a missed opportunity to initiate ART in a timely fashion.

Final 24 month treatment outcomes were available for 229 (76%) of patients started on treatment before June 2013. Among patients initiating ART, there was no significant association between time to ART initiation and final RR-TB treatment outcomes (Table 3). Treatment success was 41%, 43%, and 50% in patients who started ART within 2 weeks, between 2 to 8 weeks, and after 8 weeks respectively (p = 0.62). Patients who never started ART had a RR-TB treatment success rate of only 6% (p<0.01 compared to ever starting ART). Similarly, mortality during treatment was 21%, 13%, and 15% among patients who started ART within 2 weeks, between 2–8 weeks, and after 8 weeks(p = 0.57). However, 41% of patients who never initiated ART died during RR-TB treatment (p<0.01 compared to patients receiving ART). Of the 16 patients still on treatment without ART at four months, only two were successfully treated; two died, eight experienced loss from treatment, one failed treatment, one was transferred out with unknown outcome, and the remaining two were still receiving RR-TB treatment at time of analysis.

**Table 3:** Treatment outcomes by time to initiation of antiretroviral therapy of patients with rifampicin-resistant tuberculosis in Khayelitsha, South Africa, January 2009 –September 2012 (N = 229).

	Time to ART^a^ initiation					
Treatment Outcome	≤2 weeks	2–8 weeks	>8 weeks	Never startedART^a^	Total	P value^b^
	n (%)	n (%)	n (%)	n (%)	n (%)	
Treatment Success^c^	12 (41)	45 (43)	30 (50)	2 (6)	89 (39)	0.624
Loss from treatment^d^	8 (28)	27 (26)	15 (25)	13 (36)	63 (28)	0.948
Died^c^	6 (21)	14 (13)	9 (15)	15 (41)	44 (19)	0.570
Treatment failure^f^	0 (0)	3 (3)	3 (5)	1 (3)	7 (3)	0.613
Not evaluated^g^	3 (10)	15 (14)	3 (5)	5 (14)	26 (11)	0.177
**Total**	29 (100)	104 (100)	60 (100)	36 (100)	229(100)	

Percent (column)

*The denominator, 229, for all patients having final treatment outcome; 24 months after starting second-line treatment.

^a^ART: Antiretroviral treatment.

^b^P-value is for Fisher’s exact test for categorical values(≤2 weeks, 2–8 weeks and >8 weeks).

^c^Treatment Success: The sum of cured and treatment completed.

^d^Loss from treatment: A patient whose RR-TB treatment was interrupted for 2 consecutive months or more

^e^Died: A patient who died (all causes) during the course of treatment

^f^Treatment failure: Treatment terminated or need for permanent regimen change of at least two anti-TB drugs

^g^Not evaluated: Patients transferred to another facility to receive treatment.

**Fig 2:**
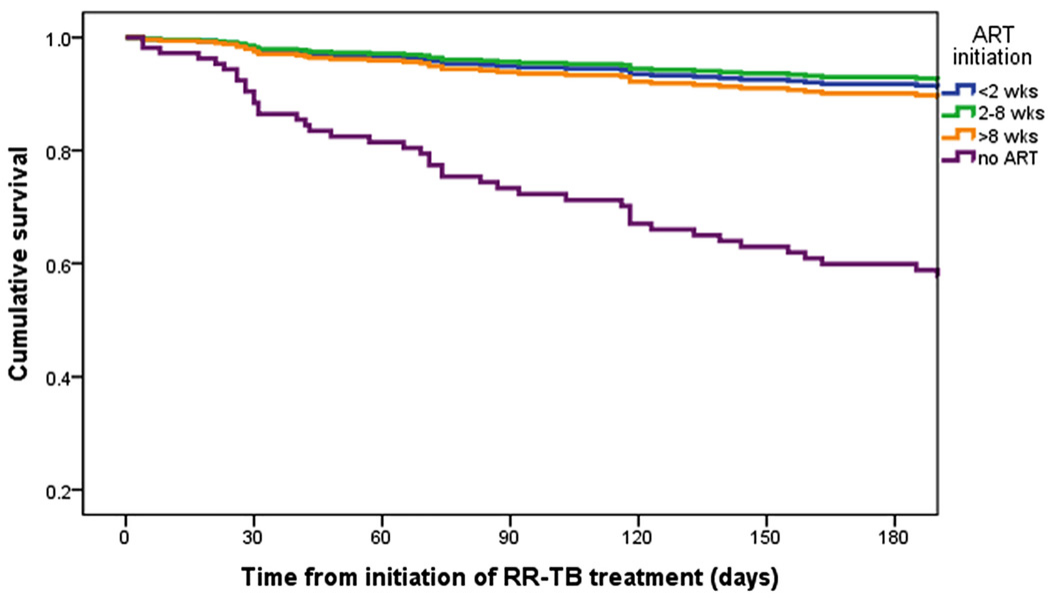
Kaplan-Meier plot of survival during treatment of rifampicin resistant tuberculosis by ART initiation, from multivariate Cox regression analysis for HIV co-infected patients.

On univariate analysis of time to death, mortality was significantly associated with not receiving ART, CD4 count <100 cells/mm^3^, and MDR-TB with either fluoroquinolone or second-line injectable resistance (Table 4). In the multivariate model, these factors remained significant. There were no significant differences in survival over six months between patients initiated on ART within 2 weeks, 2–8 weeks, and >8 weeks (Fig 2).

**Table 4:** Cox proportional hazards model of factors associated with time to death in rifampicin-resistant tuberculosis in patients co-infected with HIV and not on antiretroviral treatment at rifampicin-resistant treatment start, in Khayelitsha, South Africa, 2009–2013.

Factor	Univariate		Multivariate	
	HR^g^ (95% CI)	P value^h^	Adjusted HR^g^ (95% CI)	P value^h^
Female	0.96 (0.6–1.6)	0.86		
Age				
0–25	1.0 (Reference)			
26–30	0.85 (0.36–1.9)	0.69		
31–35	0.88 (0.4–1.9)	0.75		
36–40	0.64 (0.3–1.5)	0.3		
41+	1.0 (0.5–2.2)	0.99		
Time to ART^a^				
<2 weeks	1.0 (Reference)			
2–8 weeks	0.93 (0.3–2.7)	0.89	0.85 (0.3–2.5)	0.76
8+ weeks	0.94 (0.3–2.9)	0.91	1.2 (0.4–3.9)	0.75
No ART	5.9 (2.0–17.5)	<0.01	6.0 (2.0–18.1)	<0.01
CD4 count				
0–100	2.2 (1.1–4.4)	0.031	2.1 (1.0–4.5)	0.047
100–200	1.2 (0.5–2.8)	0.71	1.21 (0.5–3.1)	0.69
200–300	0.82 (0.3–2.4)	0.72	0.80 (0.3–2.4)	0.68
300+	1.0 (Reference)			
**RR-TB** ^b^ profile				
R-mono^c^	1.0 (Reference)			
GXP RR^d^ only	0.42 (0.1–3.1)	0.39	0.40 (0.1–3.2)	0.39
MDR^e^	0.63 (0.3–1.1)	0.14	0.82 (0.4–1.6)	0.55
MDR+SLDres^f^	2.1 (1.0–4.5)	0.049	2.5 (1.1–5.4)	0.021
**Previous TB treatment (any)**	1.8 (1.0–3.1)	0.051	1.8 (1.0–3.5)	0.059
**Year RR-TB** ^**b**^ **treatment started**				
2009	1.0 (0.5–2.1)	0.97	0.55 (0.2–1.2)	0.14
2010	0.60 (0.2–1.5)	0.26	0.47 (0.2–1.3)	0.13
2011	0.65 (0.3–1.5)	0.31	0.54 (0.2–1.4)	0.18
2012	0.64 (0.3–1.5)	0.30	0.60 (1.0–3.5)	0.25
2013	1.0 (Reference)			

Percent (column)

^a^ART: Antiretroviral treatment

^b^RR-TB: Rifampicin-resistant tuberculosis

^c^R-mono: Rifampicin mono resistance

^d^GXP: Gene-Xpert MTB/RIF rifampicin resistance only

^e^MDR: Multi-drug resistant tuberculosis with no confirmed second-line resistance

^f^SLDres: second-line drug resistance

^g^HR: Hazard ratio

^h^P value

## Discussion

Due to a paucity of data on optimal timing of ART initiation in the treatment of RR-TB, current and past guidelines concerning time to ART initiation for HIV-infected RR-TB patients in South Africa lack clarity and consensus (Table 1). Our findings show that there was a wide variation in time to ART initiation in Khayelitsha, and there were a significant proportion of patients that never started ART despite significant opportunity to do so. Among those initiated on ART there was no significant difference in either treatment success or mortality stratified by time to ART initiation.

Our data demonstrate that patients who never initiated ART have high mortality and poor outcomes overall. A small proportion of these patients died or experienced loss from treatment very rapidly after starting second-line treatment, therefore the opportunity to start ART may have been limited. However, the remainder suggest that ART initiation was either overlooked or not considered at all and is particularly concerning. While efforts have been directed at improving integration of TB and HIV services, the lack of fully integrated services is often a primary reason that RR-TB patients are not initiated on ART in a timely fashion [29,30]. Additionally, a small number of these patients were started on treatment in hospital settings and later discharged to continue treatment in the primary care setting. For these patients, continuity of care, including ART initiation, may have been compromised. Also, it is possible that some patients refused ART, particularly those with relatively high CD4 counts.

In the absence of evidence-based guidance, clinicians in Khayelitsha are likely to have used clinical judgement on timing of ART initiation among HIV infected RR-TB patients. Delays in ART initiation may have been driven by the occurrence of adverse events related to second-line treatment, particularly in the first month of treatment [10,11]. Other reasons may include concerns regarding pill burden and the impact on treatment adherence, or the risk of immune reconstitution inflammatory syndrome; patients with early ART are at more risk of developing TB-immune reconstitution inflammatory syndrome than patients with delayed ART [31–33]. In this study setting, medical records were not detailed enough to assess potential reasons for delaying ART, if this was a conscious decision by clinicians.

Overall treatment outcomes were poor, with only 39% treatment success. This is, however, comparable with national and globally reported outcomes for RR-TB programmes and recently published RR-TB/HIV co-infected patient cohort studies [1,34]. Inadequate treatment outcomes result from poorly efficacious and lengthy RR-TB treatment regimens, along with high rates of loss from treatment reported internationally, in South Africa, and within the Khayelitsha RR-TB programme [1,6,34,35].

Rapid ART initiation within two to four weeks appears to be beneficial for patients with low CD4 counts, but may not be necessary for all patients [13–15,33]. In the SAPIT trial (drug- susceptible TB patients), there was no difference in overall mortality between patients who started ART within four weeks of TB treatment initiation and those starting ART after two months of TB therapy. However, there was a survival benefit among patients with CD4 counts ≤50 cells/mm^3^ who initiated ART within four weeks [13,33]. Similar findings were demonstrated by Havlir et al [14]. Overall, there was no significant difference in the proportion of patients with an AIDS-defining illness or death between those started on ART within two weeks and those started between eight to twelve weeks after TB treatment. There was, however, a significant difference among patients with CD4 counts ≤50 cells/mm^3^ [14]. In the Cambodian CAMELIA trial, where the median CD4 count at drug-susceptible TB treatment start was less than 50 cells/mm^3^, there was a significant impact on mortality when ART was initiated two weeks after TB treatment compared to eight weeks [15].

Information regarding time to ART initiation for RR-TB patients is very limited, with only one sub-study of the SAPIT trial with 23 patients, assessing the impact of time to ART initiation for MDR-TB patients on survival with 14 patients in the combined integrated arm (ART within 12 weeks of anti-tuberculosis treatment) and nine in the sequential treatment arm (ART on completion of anti-tuberculosis drugs) [8]. In this trial starting ART early (within 12 weeks) led to a 86% reduction in mortality, whether or not patients were later switched to second-line treatment [8].A Lesotho cohort of HIV infected MDR-TB patients showed 62% success rate for ART initiation at a median of 16 days, although no comparison was made with delayed treatment initiation [7].

Our findings are consistent with those from studies of drug-susceptible TB, and suggest that delaying ART in this cohort of HIV-infected RR-TB patients, even beyond two months, does not markedly impact mortality or treatment success [13–15]. However, in our observational study the association is likely confounded by several factors. Clinicians may have based decisions on when to start ART on the clinical condition of the patient, which is known to be associated with outcomes, particularly mortality [36,37]. The direction of this association is difficult to predict, since life-saving ART may have been started more rapidly among cases with a high likelihood of poor outcomes, including very low CD4 counts. Patients who were previously treated for TB also had a tendency for decreased survival, potentially reflecting longer disease duration and worse clinical condition. While there appeared to be variation in survival by year of treatment initiation, this was not significant and therefore did not reflect the impact of changes in guideline recommendations over the study period.

In addition to the lack of documentation on reasons for timing of ART initiation, this study had limitations associated with the use of routine medical records. The study would be strengthened if data on the reasons for early or delayed ART initiation, particularly patient refusal to take ART, as well as information on previous ART history, were available. Unfortunately, medical records were not detailed enough to provide such information. Nonetheless, this was a large cohort of co-infected patients with treatment decisions reflective of routine clinical care. Given the limited number of studies assessing the impact of time to ART initiation on RR-TB patient outcomes, this study provides some additional evidence to guide clinicians regarding management of these patients.

## Conclusions

The findings of this analysis coincide with previous studies that show rapid (within 2 weeks) ART initiation for patients with low CD4 counts less than or equal to 100 cells/mm^3^ is crucial given their high early mortality rates. In addition, our results reaffirm that ART initiation is required for all RR-TB patients regardless of CD4 count due to high mortality and poor outcomes among those patients never initiated on ART. The optimal timeframe of ART initiation in the first weeks to months of RR-TB treatment remains unclear, and will likely continue to be debated amongst clinicians trying to treat this complex and high risk group of patients; the desire for timely immune restoration is unfortunately tempered by pill burden, drug interactions, and frequent toxicities of currently available drugs and RR-TB treatment regimens.
